# Genetic compensation: A phenomenon in search of mechanisms

**DOI:** 10.1371/journal.pgen.1006780

**Published:** 2017-07-13

**Authors:** Mohamed A. El-Brolosy, Didier Y. R. Stainier

**Affiliations:** Department of Developmental Genetics, Max Planck Institute for Heart and Lung Research, Bad Nauheim, Germany; Fred Hutchinson Cancer Research Center, UNITED STATES

## Abstract

Several recent studies in a number of model systems including zebrafish, *Arabidopsis*, and mouse have revealed phenotypic differences between knockouts (i.e., mutants) and knockdowns (e.g., antisense-treated animals). These differences have been attributed to a number of reasons including off-target effects of the antisense reagents. An alternative explanation was recently proposed based on a zebrafish study reporting that genetic compensation was observed in *egfl7* mutant but not knockdown animals. Dosage compensation was first reported in *Drosophila* in 1932, and genetic compensation in response to a gene knockout was first reported in yeast in 1969. Since then, genetic compensation has been documented many times in a number of model organisms; however, our understanding of the underlying molecular mechanisms remains limited. In this review, we revisit studies reporting genetic compensation in higher eukaryotes and outline possible molecular mechanisms, which may include both transcriptional and posttranscriptional processes.

## Introduction

Genetic robustness is the ability of a living organism to maintain its viability and fitness despite genetic variations, including perturbations. Genetic perturbations play an important role in evolution; however, organisms require buffering systems to ensure similar developmental outcomes despite minor differences in genetic makeup or environmental conditions, a process known as robustness or canalization [1, 2]. In 1932, dosage compensation was reported as the first example of genetic robustness. Male fruit flies were reported to have a twofold increase in transcription from their single X chromosome, resulting in the same gene expression levels as females with two active X chromosomes [3, 4]. In contrast, in mammals, females undergo inactivation of one of their X chromosomes through heterochromatization, allowing for similar developmental outcomes in both sexes [5–7]. The concept of genetic robustness was further supported by several recent studies: for example, only 20% of the protein-coding genes in yeast were reported to be essential for growth in laboratory conditions [8], and a lack of phenotype was reported for several mouse [9], zebrafish [10], and *Arabidopsis* [11] mutants.

Genetic robustness may arise from redundant genes, whereby the loss of one gene may be compensated by another with overlapping functions and expression pattern, as reported for several mutants in a range of model organisms [12–19] (reviewed in [20]). Another form of robustness arises from tightly regulated cellular networks including metabolic, signaling, and transcriptional networks. Perturbation of a particular gene’s function in a network may alter the expression of other genes within the same network, thereby maintaining cellular wellness [21, 22]. Additionally, in response to a gene knockout, organisms such as yeast may accumulate mutations in one or more genes modulating the affected pathway, thereby partially or fully rescuing the final outcome [23, 24].

While the above-mentioned modes of genetic robustness may occur as a result of the loss of function of a specific protein, a number of studies suggest a different form of genetic robustness, one that is triggered upstream of protein function (hereafter referred to as genetic compensation or transcriptional adaptation [Table 1]) [25–27]. The increasing use of recent advances in reverse genetic tools have revealed phenotypic differences between knockouts (i.e., mutants) and knockdowns (e.g., antisense-, including morpholino [MO]-, treated animals) in a number of model systems including *Arabidopsis* [28–30], mouse [31–34], *Drosophila* [35], zebrafish [10, 36], and human cell lines [37–39]. While some studies attributed these phenotypic differences to toxicity or off-target effects of the knockdown reagents [40–43] (reviewed in [44]), a recent study in zebrafish proposed gene expression changes and consequent compensation in mutant but not knockdown animals as the reason for the observed differences [25]. While knockdown of *egfl7*, an endothelial extracellular-matrix (ECM) gene, leads to severe vascular defects, most *egfl7* mutants exhibit no obvious defects. This discrepancy was attributed at least partly to the upregulation of other ECM proteins, specifically Emilins, in *egfl7* mutants but not antisense-injected embryos. Moreover, the authors observed minor or no vascular defects upon *egfl7* MO injections into *egfl7* mutants, indicating that the phenotypic differences are not due to MO toxicity. In addition, this study reported upregulation of *vegfab* mRNA levels in *vegfaa* mutant but not knockdown animals. While the mechanisms triggering the transcriptional adaptation response in *vegfaa* mutant animals remain unknown, the authors propose that it lies upstream of protein function, as overexpression of dominant-negative Vegfaa, which causes a *vegfaa* mutant-like phenotype, did not lead to an increase in *vegfab* mRNA levels. In this review, we focus on studies reporting transcriptional adaptation and/or genetic compensation in higher eukaryotes and outline possible underlying molecular mechanisms.

**Table 1:** Glossary.

Term	Definition
knockout	a genetic perturbation that aims to ablate gene function [45]
knockdown	a perturbation at the DNA, RNA, or protein level that reduces the amount of functional RNA or protein [45]
genetic compensation	changes in RNA or protein levels that can functionally compensate for the loss of function of another gene
transcriptional adaptation	changes in RNA levels that result from a genetic mutation and not from the loss of gene function

### Genetic compensation in response to gene knockout is a widespread phenomenon

Upregulation of related genes following a gene knockout may be a direct consequence of the loss of protein function. For example, mice lacking the ribosomal gene *Rpl22* show no defects in translation owing to the upregulation of its paralogue, *Rpl22l1*, the expression of which is normally inhibited by RPL22 [46]. Upregulation of related genes due to the loss of a negative feedback loop may be the first hypothesis to test when a mutant fails to show a phenotype, and a knockdown approach may help test it. For example, human *RBL2* mutant T lymphocytes proliferate normally and exhibit normal immune function due to RBL1 upregulation, an upregulation also detected upon *RBL2* knockdown in human breast cancer cell lines [47, 48], suggesting a negative feedback loop. Similarly, both knockouts and knockdowns of *HDAC-1* lead to the upregulation of HDAC-2 in several human and mouse cell lines and tissues, and vice versa [49–51].

In contrast, lack of a compensatory response in knockdown animals compared to their corresponding mutants indicates that a trigger upstream of protein function is at play, perhaps the genomic lesion itself or the mutant mRNA (Table 2). For example, small interfering RNA (siRNA)-mediated depletion of TET1, an enzyme that converts 5-methylcytosine (5mC) to 5-hydroxymethylcytosine (5hmC), in mouse embryonic stem cells (mESCs) leads to a significant reduction in 5hmC levels and a loss of undifferentiated morphology; in contrast, *Tet1* mutant mESCs exhibit only a slight decrease in 5hmC levels and maintain an undifferentiated morphology [52], suggesting possible compensation by the closely related enzyme, TET2, in mutant but not knockdown mESCs [53]. In addition, while knockdown of any of the three cyclin D family members was reported to inhibit proliferation in several cell lines [54–56], mice lacking a single isoform develop minimal defects, suggesting compensation by one of the other genes [57–59]. Indeed, knockout of two *Cyclin D* genes in mouse leads to the upregulation of the third *Cyclin D* gene. Accordingly, double knockout mice show minor phenotypes only in tissues that fail to upregulate the third *Cyclin D* gene [60]. In addition, mouse *Cyclin D2* mutant B lymphocytes exhibit no obvious proliferative phenotype due to the upregulation of *Cyclin D3* [61]. Furthermore, short hairpin RNA (shRNA)-mediated knockdown of *Importinα5* was reported to inhibit neural differentiation of mESCs cells [62]; however, *Importinα5* mutant mice display normal brain development, possibly due to the upregulation of IMPORTINα4 expression [63]. siRNA-mediated knockdown of *Kindlin-2*, which encodes an integrin coactivator, in mouse embryonic fibroblasts (MEFs) was reported to decrease INTEGRIN β1 activation and prevent INTERLEUKIN 1β–mediated increase in focal adhesion number [64]. However, *Kindlin-2* mutant cells were able to form focal adhesions due to the upregulation of KINDLIN-1 [65].

**Table 2:** Examples of discrepancies between mutant and knockdown phenotypes.

Model organism	Gene	Mutant phenotype	Knockdown phenotype	Proposed compensating gene in mutants	Reference(s)
*Arabidopsis*	*Auxin binding protein 1* (*ABP1*)	No obvious phenotype	Decreased cell expansion and division, causing a retardation in leaf growth	N/A	[28–30]
Yeast	*Bem1*	No profound defects	Defects in cell polarity and decreased cell viability	N/A	[66]
Zebrafish	*egfl7*	Minor or no vascular defects	Severe vascular defects	*emilin3a*	[25]
Mouse	*Parkin*	No mitophagy defects in mouse liver following acetaminophen (APAP) treatment	Reduced mitophagy in mouse liver following APAP treatment	N/A	[67]
	*Aqp4*	No obvious phenotype in astrocytes	Rearrangement of the filamentous actin cytoskeleton and downregulation of CX-43 in astrocytes	N/A	[68–71]
	*Tet1*	mESCs maintain an undifferentiated morphology	mESCs lose their undifferentiated morphology	*Tet2*	[52, 53]
	*Sprn*	*Sprn* and *Prnp* double mutant mice are viable.	Knockdown of *Sprn* in *Prnp* mutant mice leads to embryonic lethality.	N/A	[31, 33]
	*Ppara*	Mutant mice do not develop hypoglycemia or hypertriglyceridemia under normal feeding conditions	Knockdown mice develop hypoglycemia and hypertriglyceridemia under normal feeding conditions	N/A	[32]
	*Azi1*	No obvious phenotype in MEFs	Decreased ciliogenesis in MEFs	N/A	[26]
Human	*MELK*	No proliferation defects in several breast cancer cell lines	Decreased proliferation in several breast cancer cell lines	N/A	[72–76]

**Abbreviations:** MEFs, mouse embryonic fibroblasts; mESCs, mouse embryonic stem cells; N/A, non-applicable

In another example, antisense-mediated knockdown of *Tau* was reported to inhibit axonal elongation in cultured neuronal cells [77, 78]. However, axonal elongation was not affected in cultured neurons from *Tau* mutants, possibly due to the upregulation of microtubule-associated protein 1A (MAP1A) [79]. Interestingly, such upregulation was not detected upon *Tau* knockdown in mouse oligodendrocytes [80]. *Dystrophin* mutant mice have been reported not to develop a severe muscular dystrophy phenotype due to the upregulation of a number of genes including that encoding the dystrophin-related protein UTROPHIN [81, 82]. Interestingly, UTROPHIN upregulation was not detected in *Dystrophin* knockdown mice [83].

Furthermore, *β-Actin* mutant mice were reported to display transcriptional upregulation of several other *Actin* genes, including *γ-Actin* and *α-Actin* [27, 84, 85]. Interestingly, restoration of *β-Actin* expression in *β-Actin* mutant MEFs did not lead to a reduction in the *γ-Actin* transcriptional upregulation response, implying that this transcriptional adaptation response is triggered upstream of β-ACTIN function [27]. In addition, *γ-Actin* knockout, but not knockdown, in MEFs leads to α_sm_-ACTIN upregulation [85]. Moreover, while siRNA-mediated depletion of the centrosomal protein AZI1 in MEFs leads to a significant decrease in ciliogenesis, MEFs derived from *Azi1* mutant mice display no defects in ciliogenesis [26]. The authors also reported that *Azi1* mutant MEFs were resistant to *Azi1* siRNA, ruling out off-target effects of the siRNA and leading them to hypothesize the existence of a compensatory response in the mutant MEFs. Interestingly, this potential compensation is not observed during sperm flagella formation. This approach of testing the antisense reagent in mutant cells was subsequently used by Rossi et al. in zebrafish [25] and can be a powerful tool to identify cases of compensation in mutants versus nonspecific effects of the knockdown reagents.

### Global versus conditional loss-of-function studies

Reduction or absence of a phenotype in several germline mutants compared to their conditional counterparts has been reported in a number of studies in mouse. For example, germline mutants for *Pkm2* are viable and fertile [86]; however, conditional deletion of *Pkm2* in MEFs limits nucleotide synthesis, leading to cell-cycle arrest [87]. Similarly, *Sirt1* mutant mice have no obvious liver defects, while hepatocyte-specific *Sirt1* mutant mice develop fatty liver [88]. Mice with conditional *Fgfr3* deletion in chondrocytes exhibit more severe (and a higher incidence of) chondrona-like lesions compared to global mutant mice [89]. Moreover, conditional loss of the RETINOBLASTOMA (RB1) tumor suppressor enables cell-cycle reentry of quiescent primary MEFs, while quiescent MEFs derived from global *Rb1* mutant animals are unable to reenter the cell cycle, due at least in part to the compensatory upregulation of p107 [90]. In addition, while *Cd44* global mutant mice display only mild phenotypes [91, 92], keratinocyte-specific mutant mice display reduced epidermal stiffness and delayed wound healing, as well as reduced keratinocyte proliferation in response to 12-O-tetradecanoylphorbol-13-acetate [93]. While cell nonautonomous effects may underlie some of these discrepancies, an alternative hypothesis is that a compensatory network becomes established during germline maturation or embryonic development, allowing the organism to adapt to the mutation. Recent data in zebrafish, however, suggest that a mutation does not need to go through the germ line to induce a compensatory response [25], indicating that multiple mechanisms may underlie this process.

### Mechanisms underlying the transcriptional adaptation response

Based on the observations reported thus far, one can identify at least two possible triggers of the transcriptional adaptation response: (1) the DNA lesion and (2) the mutant mRNA. We will first speculate about how each of these potential triggers might lead to transcriptional adaptation and then briefly review other potential triggers including some that might induce posttranscriptional adaptation.

#### DNA lesion as the trigger for the transcriptional adaptation response

This section will focus on the DNA lesion being the trigger for the transcriptional adaptation response and will mostly explore the potential role of epigenetic changes following DNA damage.

**Fig 1:**
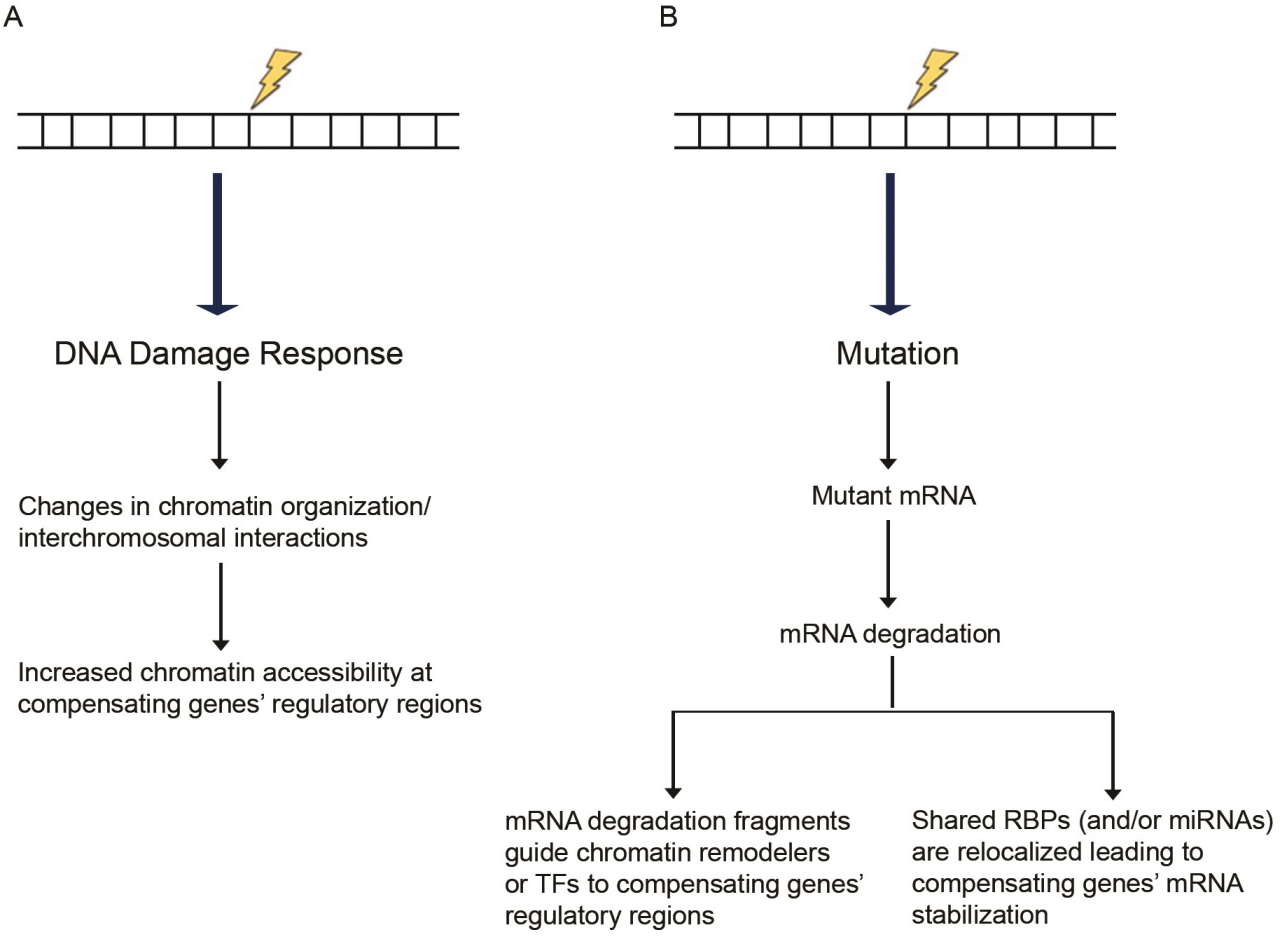
Proposed models of transcriptional adaptation. **(A)** DNA damage response can induce chromatin reorganization, increasing chromatin accessibility at the compensatory genes’ regulatory regions. **(B)** Mutations can lead to transcripts that are targeted for degradation through mRNA surveillance pathways. The resulting RNA fragments may trigger the compensatory response. As a secondary effect of the mutated gene’s mRNA degradation, RBPs or miRNAs normally acting on the mutated as well as the compensating genes’ mRNAs become more available to exert their stabilizing effects on the compensating genes’ mRNAs. **Abbreviations:** miRNAs, microRNAs; RBPs, RNA-binding proteins; TFs, transcription factors.

Following DNA damage, global chromatin reorganization and decondensation are detected [94, 95], actions mediated by several chromatin remodelers and histone-modifying enzymes (reviewed in [96]). One possibility is that in response to a mutation, global chromatin reorganization may positively affect chromatin accessibility around the compensating gene(s), thereby leading to increased expression levels (Fig 1A). Part of such a model is consistent with the process of dosage compensation in *Drosophila* where the male-specific lethal (MSL) proteins, together with other proteins, form a complex on the male X chromosome leading to H4K16 acetylation and subsequent induction of an open chromatin configuration, which is more accessible for transcription [97]. Along these lines, a *Caenorhabditis elegans* study attributed the incomplete penetrance of intestinal phenotypes in *skn-1* mutants [98] to the high variability in expression of the compensating gene *end-1* [99]. Interestingly, this variability in *end-1* expression was attributed to differences in chromatin remodeling at loci controlling *end-1* expression. It will thus be interesting to compare chromatin accessibility at the upregulated genes’ regulatory regions in wild-type, mutant, and knockdown samples.

Chromatin reorganization may be accompanied by changes in DNA looping and nuclear organization [100], which may also affect gene expression. Interchromosomal interactions are well documented (reviewed in [101]), and different kinds of stress, such as temperature, have been shown to increase interchromosomal interactions in *Drosophila* [102]. DNA damage-induced stress could similarly lead to modifications in interchromosomal interactions, including those between the mutated gene and certain other loci leading to specific gene upregulation. Chromosome-capture studies in wild-type and mutant samples to identify changes in interchromosomal interactions may help test this model.

Leading to a different potential model, a number of studies have reported the generation of small non-coding RNAs (ncRNAs) from regions spanning a double-stranded break (DSB), termed DSB-induced RNAs (diRNAs) [103, 104] (reviewed in [105]). The authors proposed that such diRNAs are essential for DNA-damage repair (DDR), possibly by acting as guides for chromatin remodelers or proteins important for DDR. Thus, diRNAs might also guide specific transcription factors (TFs) or chromatin remodelers to regulatory regions of compensating genes through homology-based interactions, leading to increased transcription. Such a model of small ncRNAs guiding specific transcription factors or chromatin remodelers to modulate gene expression is consistent with publications describing that *roX1* and *roX2* RNAs are essential for the dosage-compensation response in *Drosophila* males by guiding the assembly of the MSL protein complex on the X chromosome and subsequent histone modifications [106–108] (reviewed in [97]).

One question for these models that involve chromatin remodeling concerns the transmission of the transcriptional adaptation response to the next generation. Genomic imprinting via histone modification [109–111] (reviewed in [112]) is a possible mechanism.

In addition, induction of *GADD45A* expression following DNA damage has been reported to induce global DNA demethylation in HEK293T cells, leading to increased activation of methylation-silenced promoters [113]. Thus, one should also assess the methylation status of regulatory regions of the upregulated genes. However, to our knowledge, no links have been established to date between DNA lesions and changes in DNA methylation patterns at specific (i.e., compensating) loci.

Since all these models are based on DNA lesions, it will be important to assess transcriptional adaptation after inducing different types of mutations. One would expect the upregulation of the same genes following all types of mutations, including non-deleterious ones.

#### Mutant mRNA as the trigger for the transcriptional adaptation response

This section will focus on the mutant mRNA being the trigger for the transcriptional adaptation response. After reviewing a few examples, we will focus specifically on how RNA fragmentation by different mRNA surveillance pathways could trigger such a response.

Mutations often lead to mRNAs with a premature termination codon (PTC), secondary structures that stall ribosomal translocation, or, less frequently, mRNAs that lack a stop codon. The presence of such mRNAs triggers the nonsense-mediated decay (NMD), no-go decay, or no-stop decay pathways, respectively, which results in mRNA degradation (reviewed in [114–116]). A recent study in zebrafish reported that two different mutations in the same exon of *mt2* cause different degrees of phenotypic severity. Surprisingly, the mutant allele with the milder phenotype exhibited a higher degree of NMD. Antisense-mediated knockdown of the NMD pathway and consequent decrease in mutant mRNA degradation led to a more severe phenotype, consistent with the possibility that NMD triggers a compensatory response that decreases the severity of the mutant phenotype [117]. One hypothesis is that the RNA fragments resulting from the mRNA surveillance pathways function to regulate gene expression. While the current understanding in the field is that the mRNA surveillance pathways lead to processive mRNA degradation [114, 118], it is possible that short-lived and relatively rare degradation intermediates are present.

If the fragments are long enough, one can hypothesize that they act in a fashion similar to long noncoding RNAs (reviewed in [119]) and, for example, guide specific transcription factors or chromatin remodelers to the regulatory regions of compensating genes through homology-mediated base pairing (Fig 1B). Other studies have reported that injection of short (20–22 nt) RNA fragments from a specific mRNA leads to increased transcription of the corresponding locus [120, 121]. Mechanistically, the authors report that the injected sense RNA fragments can form double-stranded RNA (dsRNA) duplexes with short antisense transcripts normally produced from the locus. The resulting dsRNAs may then be utilized by the RNA interference (RNAi) machinery in an ARGONAUTE-dependent manner to induce chromatin modifications at the locus and increase euchromatin histone marks or decrease heterochromatin histone marks. Although the exact machinery underlying such dsRNA-induced epigenetic changes remains unknown, this model is consistent with several other studies reporting transcriptional activation through histone modification following targeting of dsRNA to the promoter region of various genes [122–125]. Previous analyses of the mouse and human transcriptome have identified several antisense transcripts that can participate in forming sense/antisense pairs [126–130]. Thus, one could hypothesize that RNA fragments act in a similar fashion and form dsRNA duplexes with antisense transcripts from the compensating loci, leading to transcriptional upregulation.

RNA-binding proteins (RBPs) can also regulate gene expression in a number of ways (reviewed in [131]), one of which is by increasing gene expression through stabilizing mRNAs [132]. The highly dynamic binding of RBPs is regulated by cellular conditions; therefore, regulating RBP interactions following genotoxic stress may be a mechanism for the cell to compensate for a lost gene. Along these lines, mRNAs that encode functionally related proteins tend to be coregulated by specific RBPs, forming what is known as RNA operons or RNA regulons [133–135] (reviewed in [136]). Thus, the mutant and compensating genes might be regulated by the same RBPs, and if the mutant mRNA is subjected to degradation or if its secondary structure is affected by the mutation (thereby affecting RBP binding), RBPs would become available to stabilize the compensatory genes’ mRNAs (Fig 1B).

Besides their well-known function in silencing gene expression [137], micro-RNAs (miRNAs) can enhance gene expression through several mechanisms. Although miRNAs normally target mRNAs, *miRNA-373* was reported to bind promoter regions of *CDH1* and *CSDC2* in PC3 (a human prostate cancer cell line) cells and induce their expression through an unknown mechanism [138]. miRNAs can also increase the translation of certain mRNAs; for example, under amino acid starvation conditions, *miRNA10a* was reported to bind the 5′UTR of ribosomal protein mRNAs and enhance their translation [139]. miRNAs have multiple target mRNAs [140, 141], and, thus, if a mutation leads to mRNA degradation, the miRNAs targeting the affected gene will become available to modulate other targets (Fig 1B).

Since these models rely on the generation and potential degradation of mRNAs from the mutated locus, one would not expect upregulation of potentially compensating genes in the absence of active transcription of the mutant mRNA. It will thus be important to assess transcriptional adaptation in alleles where an mRNA is not produced.

#### Other potential mechanisms for the compensatory response

This brief section will focus on increased translational response following the mutational loss of specific genes and will evoke processes such as mRNA modifications and upstream open reading frames.

In response to stress (such as heat shock), pseudouridylation or N6-methylation of adenosines (m6A) was reported to be enriched on certain mRNAs, thereby increasing their stability or promoting their translation [142, 143] (reviewed in [144]). However, as is the case for DNA methylation, there has been no report thus far about mRNAs from selective loci being modified in this manner.

Upstream open reading frames (uORFs) are regulatory elements present in the 5’UTRs of around 50% of vertebrate mRNAs [145, 146]. They may act as translational repressors, as the translation of the uORFs can occur at the expense of that of the mRNA’s coding sequence [147, 148]. Under cellular stress conditions, there is a tendency to inhibit global translation by phosphorylating eIF2α, which then acts as a competitive inhibitor of the translation initiation factor eIF2B, thereby reducing translation reinitiation rates [149]. This mechanism may allow the increased translation of certain mRNAs under cellular stress. For example, the yeast transcription factor gene *GCN4* has 4 uORFs and under normal conditions, the 4 uORFs are translated with less reinitiation at the main ORF. Under nutritional stress, the first uORF is translated efficiently; however, due to eIF2α phosphorylation, the remaining uORFs are poorly translated, and reinitiation only occurs at the main ORF, thereby increasing GCN4 production [150]. It is thus possible that certain gene mutations induce cellular stress, allowing for uORF skipping and increased translation of compensating genes. However, as is the case for the DNA and RNA methylation modifications mentioned above, it is not clear how specificity, in terms of selective proteins being upregulated, would arise.

## Conclusion

Despite its role in maintaining an organism’s robustness, the molecular mechanisms underlying genetic compensation remain poorly understood. Here, we reviewed studies reporting genetic compensation in several higher eukaryotes, outlined potential underlying mechanisms, and proposed experiments that should help test these potential mechanisms. Studying epigenetic changes following DNA damage, a major difference between mutants and knockdowns, should allow a better understanding of why a compensatory response is triggered by knockout but not knockdown approaches. Moreover, we also proposed mRNA surveillance pathways, ncRNAs, uORFs, RBPs, and miRNAs as potential players in the compensatory response.

Recently, a study of more than 500,000 human genomes identified 13 individuals harboring disease-causing mutations in 8 different genes, with no reported clinical manifestation of the disease [151]. Other studies on Icelandic and British people identified complete gene knockouts in several apparently healthy individuals [152, 153]. While functional characterization of the identified alleles still remains to be completed, it is likely that genetic compensation underlies the lack of phenotype in individuals with severe mutant alleles. Moreover, several factors have been proposed to explain the concept of incomplete penetrance, including environmental factors, different genetic backgrounds, and different expression levels of modifier genes [154, 155]; however, this concept remains poorly understood, as a recent study reported that incomplete penetrance is even common in mice with the same genetic background [156]. We propose that incomplete penetrance may be due to compensatory responses being triggered in some individuals but not in others. Investigating the molecular mechanisms underlying genetic compensation may help us understand why some mutations cause disease while others do not. It might also lead to the development of more effective therapies that enhance an organism’s robustness to a mutation rather than correct its effect, e.g., increase the expression of the compensating gene(s) rather than correct the function of the defective gene.
